# Predictors of Seronegative Conversion After Centralized Management of Syphilis Patients in Shenzhen, China

**DOI:** 10.3389/fpubh.2021.755037

**Published:** 2021-11-25

**Authors:** Zhenzhou Luo, Yi Ding, Jun Yuan, Qiuhong Wu, Lishan Tian, Li Zhang, Bo Li, Jinsong Mou

**Affiliations:** ^1^Shenzhen Nanshan Center for Chronic Disease Control, Shenzhen, China; ^2^Pingshan District Maternal and Child Healthcare Hospital of Shenzhen, Pingshan General Hospital of Southern Medical University, Shenzhen, China

**Keywords:** syphilis, seroconversion, Shenzhen, China, multivariable analysis, TRUST titer, centralized management, STIs

## Abstract

**Objective:** The aim of this study was to explore the seronegative conversion status of syphilis patients after centralized management and to analyze potential determinants.

**Materials and Methods:** A retrospective population-based cohort study was conducted, and data for individuals who had been diagnosed with syphilis between 2011 and 2019 were retrieved from the Shenzhen Nanshan Center for Chronic Disease Control. Seroconversion statuses were summarized as percentages. Univariable and multiple Cox proportional hazard regression models were used to analyze the factors associated with seronegative conversion among syphilis patients.

**Results:** During the study period, 1,545 patients with syphilis participated in the syphilis convergence case management program on a voluntary basis, of whom 290 were excluded due to missing follow-up data. A total of 27.6% (346/1255) of patients with syphilis showed seronegative conversion. Multivariable analysis revealed that the following significantly determined syphilis seroconversion from positive to negative: younger age (15–19 years vs. ≥30 years: HR = 2.18), male gender (HR = 1.45), lower baseline toluidine red unheated serum test (TRUST) titer of ≤ 1:8 (HR = 2.23), and different disease stages, including latent syphilis (HR = 1.98), primary syphilis (HR = 7.67), and secondary syphilis (HR = 4.83).

**Conclusions:** Few patients with syphilis tested negative after treatment at the end of the study. Seronegative conversion in the patients was associated with age, sex, baseline TRUST titer, and syphilis stage.

## Introduction

Syphilis is a sexually transmitted infection (STI) caused by *Treponema pallidum* and is spread through contact with infectious lesions or body fluids (1). Patients typically develop cutaneous manifestations such as genital ulcers and various complications, including neurologic, renal, gastrointestinal, and hepatic diseases (2).

Despite the availability of inexpensive and effective antibiotic therapy, syphilis remains a prevalent disease in developing countries and has re-emerged as a public health threat in developed nations. Syphilis has an estimated global prevalence of 36 million cases and an incidence of over 11 million cases annually (3). In the South African adult (15–49 years) population in 2017, the estimated prevalence of syphilis among women and men was 0.50% (95% CI: 0.32–0.80%) and 0.97% (0.19–2.28%), respectively (4). In the United States, from 2013 to 2017, the national annual rate of reported primary and secondary (P&S) syphilis cases increased by 72.7%, from 5.5 to 9.5 cases per 100,000 individuals (5). A review of syphilis studies in Eastern Europe showed that even though the incidence was generally declining, high prevalence was reported in key populations, particularly sex workers and users of injectable drugs (6). In China, a range of unique biological and social forces, such as depletion of individuals with immunity, income gaps and a cultural climate that favors the re-emergence of sex work, are driving the spread of syphilis (7). With the current economic and social developments, the prevalence of sexually transmitted disease (STD) remains an important concern.

To control the disease, China has continually implemented massive syphilis control programs. However, during the past 20 years, syphilis has resurged in China (7), with a significant increase in incidence from 2003 to 2013 (8, 9). The syphilis burden has been greatest in coastal urban China areas, such as Guangdong Province (10). A meta-analysis showed that Guangdong was among the provinces of China with a very high syphilis prevalence of more than 10% (11). A significant rise in syphilis cases has been observed in Guangdong Province over the last decade (12). From January 2014 through June 2015, 82,554 syphilis cases were reported in this province. Concurrently, syphilis spatial clustering was found in the city of Shenzhen (13). A total of 6,807 syphilis cases were reported in 2013 in Shenzhen (14). The burden of syphilis in Shenzhen is large, and syphilis control should therefore be regarded as a public health priority.

Although some researchers have reported on seroconversion and its determinants after treatment (15–17), studies on syphilis seronegative conversion and associated factors in Shenzhen are limited. Generally, patients with effective treatment for syphilis have been defined as patients having a ≥4-fold decreased titer after treatment during follow-up. However, some patients do not exhibit seronegative conversion but rather enter a serofast state. Although seroconversion from positive to negative is recognized as the final goal of treatment for doctors and patients, some studies have found that a serofast state can also reduce the psychological burden of patients. Seronegative conversion means that syphilis has been cured completely, without risk of recurrence; thus, it is an important index of effective treatment. Therefore, this study was conducted to explore post-syphilis seronegative conversion predictors. The results can facilitate the identification of high-risk populations and promote treatment outcomes. Moreover, they can provide a scientific basis for the centralized management of syphilis. In April 2011, Nanshan District, Shenzhen, launched the Syphilis Convergence Case-Management Program to consolidate prevention, treatment, and management for better syphilis control. All health organizations in the district were asked to refer patients with syphilis seropositivity to the STD clinic of the Department of Dermatology and Venereology in Nanshan Center for Chronic Disease Control, where patients were subject to centralized management, standardized treatment, and regular serologic follow-up. By running this program, we aimed to observe the outcome of seronegative conversion after the centralized management of patients with syphilis and to explore potentially associated factors.

## Materials and Methods

### Study Population

A retrospective population-based cohort study was conducted. We retrospectively analyzed the data from syphilis patients managed in the clinic of Shenzhen Nanshan Hospital for Chronic Diseases between 2011 and 2019. Patients who met the following criteria were included in the study: (1) diagnosed with syphilis, (2) had related information about treatment, and (3) completed at least one serological follow-up visit after treatment. People with any of the following conditions diagnosed by a doctor were excluded: severe heart, liver, or kidney disease; malignant disease; chronic infectious disease, such as tuberculosis, leprosy, or viral hepatitis; systemic autoimmune disease, such as lupus erythematosus, rheumatoid arthritis, or dermatomyositis; and severe mental illness.

### Diagnosis, Treatment, and Follow-Up

The patients were diagnosed with syphilis infection according to epidemiological history, clinical signs and symptoms, and toluidine red unheated serum test (TRUST)/*Treponema pallidum* particle agglutination (TPPA) results. Some patients actively underwent syphilis testing when they had venereal symptoms or had engaged in high-risk behavior. Syphilis infection in other patients was discovered through passive testing, such as physical examinations, blood donation and preoperative examination. Non-penicillin-allergic participants were treated with benzathine penicillin G (BPG) in one or two courses (2.4 million units BPG weekly for 2 weeks as one course). Penicillin-allergic participants received doxycycline (100 mg taken orally twice daily for 14 days). All participants were notified of the need for follow-up for 1 year after treatment. Quantitative and qualitative testing of TRUST, indicating serological conversion negativity or lack thereof, was performed during follow-up, and the resolution of clinical signs and symptoms was evaluated.

### Data Collection

Qualified professional STD physicians were installed as program investigators to collect data. They underwent unified training and became familiar with a written investigation manual prior to data collection. Face-to-face interviews were conducted with a designed questionnaire to obtain related information from the patients at the beginning of the study. Written patient consent was obtained according to institutional guidelines.

### Laboratory Testing

Blood specimens were tested for syphilis. TRUST (Rongsheng Biotech Company, Shanghai, China) and TPPA tests (Fujirebio, Tokyo, Japan) were performed on serum samples. The operational instructions were strictly obeyed, and the outcomes were identified by multiple researchers.

### Statistical Analysis

The information was recorded in a database using EpiData 3.0 and analyzed using SPSS 25.0. Descriptive statistics and chi-square tests were used to analyze the differences in seronegative conversion of different syphilis subpopulations. The endpoint was defined as the outcome of the TRUST test becoming negative. If the endpoint was not reached, the patient was defined as censored. If someone was lost to follow-up, the final follow-up result was used. Univariable and multiple Cox proportional hazard analyses were performed to determine the factors associated with seronegative conversion among the patients with syphilis. Variables in the univariable analyses with a *p*-value < 0.30 were included in subsequent multivariable regression models. Multiple regression models were built using stepwise techniques. The selection of variables in the final model was conducted using a forward-conditional method, with significance levels of ≤ 0.05 for inclusion and ≥0.1 for exclusion. A *p*-value ≤ 0.05 was considered statistically significant.

## Results

### Characteristics of the Patients

During the study period, 1,545 patients with syphilis received routine syphilis management; 290 were excluded due to missing follow-up data. Finally, a total of 1,255 eligible patients with syphilis were included. The average observation time was 9.5 ± 3.2 months. A total of 750 (59.8%) and 505 (40.2%) patients were male and female, respectively, corresponding to a sex ratio of 1.5:1. The mean age was 35.9 ± 11.9 years, and the ages ranged from 15 to 89 years. There were 490 (39.0%) unmarried patients, 673 (53.6%) married patients, and 92 (7.3%) patients who were divorced or widowed. There were 651 (51.9%) patients with latent syphilis, 113 (9.0%) patients with primary syphilis, 249 (19.8%) patients with secondary syphilis, and 242 (19.3%) patients receiving adequate treatment.

### Outcomes of Seronegative Conversion Among Syphilis Patients

Table 1 shows the outcome of seronegative conversion among the patients. According to the TRUST results, the total percentage of seronegative conversions among the patients was 27.6% (346/1255).

**Table 1:** Outcomes of TRUST seronegative conversion among syphilis patients.

	**N**	**TRUST seronegative conversion (n, %)**	**χ^2^**	***P***
**Age (years)**			2.605	0.272
15~19	32	12 (37.5)		
20~29	427	124 (29.0)		
≥30	796	210 (26.4)		
Sex			41.861	<0.01
Male	750	257 (34.3)		
Female	505	89 (17.6)		
Marital status			4.966	0.084
Unmarried	490	152 (31.0)		
Married	673	169 (25.1)		
Divorced/Widowed	92	25 (27.2)		
Method of syphilis discovery			22.226	<0.01
Active detection	897	281(31.3)		
Passive detection	358	65(18.2)		
Initial TRUST titer			5.253	0.022
≤ 1:8	711	214 (30.1)		
>1:8	544	132 (24.3)		
Syphilis stage			178.673	<0.01
Latent	651	136 (20.9)		
Primary	113	84 (74.3)		
Secondary	249	95 (38.2)		
After adequate treatment	242	31 (12.8)		
Treatment regimen			18.776	<0.01
Untreated	159	22 (13.8)		
One course of BPG	928	281 (30.3)		
Two courses of BPG	134	34 (25.4)		
Replacement therapy with doxycycline	34	9 (26.5)		
People living with HIV			1.978	0.159
Yes	148	48 (32.4)		
No	1,107	298 (26.9)		
Male homosexuality			3.361	0.067
Yes	174	58 (33.3)		
No	1,081	288 (26.6)		
Bisexuality				<0.01
Yes	41	22 (53.7)		
No	1,214	324 (26.7)		

At the end of the study, ~257 (34.3%) male patients with syphilis tested negative, which was more than the number of female patients (*n* = 89, 17.6%) (*p* < 0.01). Patients who adopted the syphilis test actively showed a higher frequency of TRUST seroconversion from positive to negative than those who received the test passively (*n* = 281, 31.3% vs. *n* = 65, 18.2%, *p* < 0.01).

Patients with a baseline TRUST titer of ≤ 1:8 had a higher frequency of seronegative conversion than those with a TRUST titer of >1:8 (*n* = 214, 30.1% vs. *n* = 132, 24.3%, *p* = 0.022). Eighty-four (74.3%) patients with primary syphilis underwent TRUST seronegative conversion, with a higher frequency of conversion observed in these patients than in patients with secondary syphilis (*n* = 95, 38.2%), patients with latent syphilis (*n* = 136, 20.9%), and patients who were receiving adequate treatment (*n* = 31, 12.8%) (*p* < 0.01). Patients with one course of BPG had a higher frequency of TRUST seronegative conversion (*n* = 281, 30.3%) than those with no treatment (*n* = 22, 13.8%), two courses of BPG (*n* = 34, 25.4%), or replacement therapy with doxycycline (*n* = 9, 26.5%). A higher frequency of TRUST seronegative conversion was found in bisexual patients with syphilis (*n* = 22, 53.7%) than in heterosexual patients (*p* < 0.01). TRUST seronegative conversion for syphilis was not significantly associated with age, marital status, HIV infection, or male homosexuality (*p* > 0.05).

### Factors Associated With Seronegative Conversion Among Syphilis Patients

Univariable Cox proportional hazard regression analyses were performed to identify the factors associated with seronegative conversion. Male patients with syphilis were more likely to test seronegative than were female patients (HR = 1.88, 95% CI = 1.48–2.39; *p* < 0.01). Patients who had actively undergone the syphilis test were more likely to show seronegative conversion than those who had undergone the test passively (HR = 1.61, 95% CI = 1.23–2.11, *p* < 0.01). Furthermore, patients with a lower initial TRUST titer of ≤ 1:8 were more likely to test negative than those with an initial TRUST titer of >1:8 (HR = 1.34, 95% CI = 1.08–1.67, *p* = 0.01). Compared to patients who were receiving adequate treatment, patients with latent syphilis (HR = 1.84, 95% CI = 1.24–2.71, *p* < 0.01), primary syphilis (HR = 7.60, 95% CI = 5.03–11.48, *p* < 0.01), and secondary syphilis (HR = 3.18, 95% CI = 2.12–4.76, *p* < 0.01) were more likely to show seronegative conversion. Compared to untreated patients, those receiving treatment regimens, including one course of BPG (HR = 2.38, 95% CI = 1.54–3.67, *p* < 0.01), two courses of BPG (HR = 1.84, 95% CI = 1.08–3.14, *p* = 0.03), and replacement therapy with doxycycline (HR = 2.26, 95% CI = 1.04–4.90, *p* = 0.04), were more likely to test negative. Male homosexual individuals were more likely to show seronegative conversion (HR = 1.36, 95% CI = 1.03–1.80, *p* = 0.03) and bisexual syphilis patients (HR = 1.71, 95% CI = 1.11–2.64, *p* = 0.01) were associated with syphilis TRUST seroconversion from positive to negative (Table 2).

**Table 2:** Results of univariable and multivariable analyses of factors associated with TRUST seronegative conversion among syphilis patients.

	**Univariable analysis**				**Multivariable analysis**			
	**b**	**Wald χ2**	***P***	**Hazard ratio (95% CI)**	**b**	**Wald χ2**	***P***	**Hazard ratio (95% CI)**
Age (years)		2.66	0.27			6.82	0.03	
15~19	0.44	2.22	0.14	1.56 (0.87, 2.79)	0.78	6.71	0.01	2.18 (1.21, 3.94)
20~29	0.10	0.75	0.39	1.10 (0.88, 1.38)	0.01	0.01	0.93	1.01 (0.80, 1.27)
≥30				1				1
Sex
Male	0.63	26.34	<0.01	1.88 (1.48, 2.39)	0.37	7.72	0.01	1.45 (1.12, 1.88)
Female				1				1
Marital status		4.30	0.12					
Unmarried	0.10	0.22	0.64	1.11 (0.73, 1.69)				
Married	−0.13	0.37	0.54	0.88 (0.58, 1.34)				
Divorced/Widowed				1				
Method of syphilis discovery								
Active detection	0.48	11.92	<0.01	1.61 (1.23, 2.11)				
Passive detection				1				
Initial TRUST titer								
≤ 1:8	0.29	7.02	0.01	1.34 (1.08, 1.67)	0.80	36.77	<0.01	2.23 (1.72, 2.88)
>1:8				1				1
Syphilis stage		142.04	<0.01			125.75	<0.01	
Latent	0.61	9.30	<0.01	1.84 (1.24, 2.71)	0.68	11.73	<0.01	1.98 (1.34, 2.93)
Primary	2.03	93.00	<0.01	7.60 (5.03, 11.48)	2.04	87.72	<0.01	7.67 (5.01, 11.74)
Secondary	1.16	31.22	<0.01	3.18 (2.12, 4.76)	1.58	48.02	<0.01	4.83 (3.09, 7.54)
After adequate treatment				1				1
Treatment regimen		16.48	<0.01					
Untreated				1				
One course of BPG	0.87	15.31	<0.01	2.38 (1.54, 3.67)				
Two courses of BPG	0.61	4.94	0.03	1.84 (1.08, 3.14)				
Replacement therapy with doxycycline	0.81	4.22	0.04	2.26 (1.04, 4.90)				
People living with HIV								
Yes	0.13	0.68	0.41	1.14 (0.84, 1.54)				
No				1				
Male homosexuality								
Yes	0.31	4.57	0.03	1.36 (1.03, 1.80)				
No				1				
Bisexuality								
Yes	0.54	5.99	0.01	1.71 (1.11, 2.64)				
No				1				

All variables at *p* < 0.3 in the univariable analyses were included in the multivariable Cox regression analysis. A forward selection (conditional) method was conducted to select variables, with a significance level of ≤ 0.05 required for inclusion and of ≥0.1 required for exclusion. The multivariable analysis suggested that patients of younger age (15–19 years vs. ≥30 years: HR = 2.18, 95% CI = 1.21–3.94, *p* = 0.01) and male gender (HR = 1.45, 95% CI = 1.12–1.88, *p* = 0.01) were more likely to test negative. Patients with a lower initial TRUST titer of ≤ 1:8 more easily underwent TRUST seroconversion from positive to negative than those with a TRUST titer of >1:8 (HR = 2.23, 95% CI = 1.72–2.88). Moreover, compared to patients who were receiving adequate treatment, patients at different disease stages, including latent syphilis (HR = 1.98, 95% CI = 1.34–2.93, *p* < 0.01), primary syphilis (HR = 7.67, 95% CI = 5.01–11.74, *p* < 0.01), and secondary syphilis (HR = 4.83, 95% CI = 3.09–7.54), were significantly more likely to show syphilis TRUST seroconversion (Table 2).

## Discussion

Syphilis is a persistent public health issue in many countries. Its prevalence has recently increased in some countries (18, 19). In Guangdong Province, the syphilis incidence rate increased yearly between 2005 and 2014—from 21.08/100,000 to 52.55/100,000 over this period (20). A systematic review found that 90% of syphilis patients were from resource-limited countries (21). Therefore, the Syphilis Convergence Case-Management Project was implemented to promote prevention and control. Through our research, the state of seronegative conversion and a few significant factors associated with seronegative syphilis conversion were discovered. The research results provide some basis for building an effective and synthetic model of syphilis prevention and control.

Only 27.6% of the patients showed TRUST seronegative conversion at the end of our study. A previous study reported that the rate of syphilis serological cure (17, 22) was 65–79%; however, the sample size was smaller than that in the present study. Moreover, some of the patients in our study originated from other health care facilities, and their past medical history and treatments were comparatively complicated. In addition, treatment cost, treatment adherence, and type of health care system might be correlated with seroconversion. The percentage of seronegative conversion in our study also suggests that the level of management of syphilis convergence cases, such as treatment, follow-up, and data collection, needs to be strengthened in the future.

In our study, we found that seronegative conversion was independently associated with younger age, as was also reported by Seña et al. (17), who revealed that the probability of achieving serological cure decreased with age. This association may be related to the senescence of the immune system at older age, which can be expected to influence the serological response to syphilis therapy (22). In addition, more male patients tested negative for TRUST than female patients (34.3% vs. 17.9%). The multivariable analysis also showed that male patients with syphilis were more likely to test seronegative. This difference might be related to the differences in the immune system between men and women (23), which influence the serological response. The exact mechanism underlying the association with sex is still unclear and needs further investigation.

Understanding the relationship of quantitative non-treponemal titers with disease is vital for evaluating treatment response, which can reflect the activity of the disease process or the immune response. Generally, non-treponemal antibody titers are related to disease activity. However, some studies demonstrated that patients with higher baseline rapid plasma reagin (RPR) titers were more likely to achieve serological cure (17, 22). Baker-Zander et al. (24) found that Venereal Disease Research Laboratory (VDRL)-immunized rabbits exhibited partial protection against reinfection with *T. pallidum*, which meant that high VDRL antibody titers might help control the infection. However, in our study, the patients with lower initial TRUST titers easily became seronegative. The multivariable model also revealed that the likelihood of TRUST seronegative conversion was increased with a decrease in the initial TRUST titer. There may be a negative association between the TRUST titer and the quantity of *T. pallidum*. The detailed mechanism requires clarification through further studies. Hence, the level of syphilis antibody titer might correspond to the levels of syphilis and immune response. Facilitating routine syphilis serological tests and follow-up in the clinic is integral to observing the efficacy of treatment and preventing adverse outcomes.

We also found that patients with primary syphilis were more likely to experience TRUST seronegative conversion than those who were receiving adequate treatment before enrollment and more so than patients with secondary and latent syphilis. The results of the current study were consistent with a previous observation that patients with secondary syphilis were more likely to experience treatment failure than those with primary syphilis (25). In addition, Tong et al. reported that the rate of serological cure decreased in the order of primary, secondary, latent, and tertiary syphilis (22). The differences in immunological functions among different disease stages may be responsible for this observation. These results suggest that enhancing early diagnosis, treatment, and city-specific syphilis screening strategies is very important and that the clinical stages of syphilis often overlap.

## Limitations

Our study has several limitations. First, there may be some selection bias because several patients were excluded due to missing data. Second, the results correspond to only one district and might not be generalizable to other areas; thus, the representativeness of the sample needs to be improved. Third, some reporting bias may have been inherent in this study because some subjects may have hidden sensitive information.

## Conclusion

The present study showed that syphilis TRUST seronegative conversion after treatment was poor. However, syphilis seroconversion from positive to negative was significantly associated with sex, baseline TRUST titer, and disease stage.

## Data Availability Statement

The raw data supporting the conclusions of this article will be made available by the authors, without undue reservation.

## Ethics Statement

The studies involving human participants were reviewed and approved by Shenzhen Nanshan Center for Chronic Disease Control. Written informed consent to participate in this study was provided by the participants' legal guardian/next of kin.

## Author Contributions

ZL and BL conceived and designed the study. JY and QW implemented the study and conducted data collection. LT and LZ contributed to data analysis. ZL and YD wrote and drafted the manuscript. JM was involved in critical revision of the manuscript. All authors have read and approved the final manuscript.

## Funding

The Shenzhen Healthcare Research Project (SZGW2018001) supported this study.

## Conflict of Interest

The authors declare that the research was conducted in the absence of any commercial or financial relationships that could be construed as a potential conflict of interest.

## Publisher's Note

All claims expressed in this article are solely those of the authors and do not necessarily represent those of their affiliated organizations, or those of the publisher, the editors and the reviewers. Any product that may be evaluated in this article, or claim that may be made by its manufacturer, is not guaranteed or endorsed by the publisher.

## Abbreviations

STI: sexually transmitted infection
TRUST: toluidine red unheated serum test
HR: hazard ratio
CI: confidence interval
STD: sexually transmitted disease
TPPA: *Treponema pallidum* particle agglutination
BPG: benzathine penicillin G
RPR: rapid plasma reagin.
